# *Staphylococcus aureus*: Screening for Nasal Carriers in a Community Setting with Special Reference to MRSA

**DOI:** 10.1155/2014/479048

**Published:** 2014-06-25

**Authors:** Yukti Sharma, Sanjay Jain, Harshvardhan Singh, Vasudha Govil

**Affiliations:** ^1^Department of Microbiology, Hindu Rao Hospital, Delhi 110007, India; ^2^Department of Biochemistry, Hindu Rao Hospital, Delhi 110007, India

## Abstract

*Introduction.* Emergence of MRSA infections among previously healthy persons in community settings (without exposure to health care facilities) has been noted recently. MRSA infections are now classified as health care-associated MRSA (HA-MRSA) and community-associated MRSA (CA-MRSA) infections. Its colonization is an important risk factor for subsequent MRSA infection. *Aims and Objectives.* The aim was to screen patients and health care workers for staphylococcal carriage, identify risk factors for MRSA colonization, and determine the sensitivity pattern. *Materials and Methods.* A total of 200 subjects were screened for nasal carriage after obtaining verbal consent. These were both healthy subjects attending various outpatient departments and health care workers. Specimens were collected from the anterior nares using premoistened sterile cotton swabs and inoculated onto blood agar and mannitol salt agar and incubated at 37°C for 24–48 h. *Results. Staphylococcus aureus* colonisation was found to be 12% (*n* = 24). MRSA was identified in 5% (*n* = 10) which represents 41.66% of SA. A total of 10 strains of MRSA were isolated from 200 subjects, giving an overall positivity rate of 5%. *Discussion.* Staphylococcal colonization was found to be 12% (MRSA 5%). Fluoroquinolone resistance was remarkable whereas all strains were sensitive to vancomycin, teicoplanin, linezolid, quinupristin-dalfopristin.

## 1. Introduction

The emergence of MRSA infections among previously healthy persons in community settings (without exposure to health care facilities) has been noted recently [1, 2]. Therefore, MRSA infections are now classified as health care-associated MRSA (HA-MRSA) infections and community-associated MRSA (CA-MRSA) infections [3]. Apparently acquired in the community, these infections are termed as community-associated MRSA infections (CA-MRSA) [4, 5]. CA-MRSA strains and health care-associated (HA) MRSA strains differ in terms of epidemiology, microbiology, and clinical manifestations [5]. Colonization rates of MRSA in the community have been reported to range from 0 to 9.2 percent [6]. The nose and open skin areas (i.e., wounds and device exit sites) are considered the most important sites for colonization [7–10]. Nasal carriage of MRSA is an important risk factor for subsequent MRSA infection and transmission of this pathogen [11]. Active surveillance for patients colonized with methicillin resistant* Staphylococcus aureus* (MRSA) is recommended to prevent MRSA infections in health care settings [7, 12]. Although several studies have reported the prevalence of MRSA nasal carriage in patients in health care settings [13, 14], this subject has been little investigated in healthy individuals in the broader community [15]. Infection control teams in local Delhi hospitals expressed concern that an increasing number of patients were being admitted to hospital already colonized with MRSA. It was suggested that there might be a high prevalence of MRSA in the local community and so we undertook a prevalence survey of MRSA colonization in the general population in Delhi. The objectives of the present study were to screen patients and health care workers for MRSA carriage, to identify risk factors for MRSA colonization, and to determine their resistance pattern. CA-MRSA is responsible for around 30 percent of* S. aureus* infections in hospitals of USA [16].

There is a growing urgency to study the colonization behaviour of CA-MRSA.

## 2. Materials and Methods

A total of 200 subjects (170 outpatients and 30 health care workers) were screened for MRSA after obtaining verbal consent from subjects. Using premoistened sterile cotton swabs, specimens were collected from the anterior nares of patients and health care workers.

The specimens were inoculated onto sheep blood agar and mannitol salt agar and incubated at 37°C in ambient air for 24–48 h. Colonies suggestive of* S. aureus* (white or cream haemolytic or nonhaemolytic colonies on oxacillin blood agar and yellow colonies on mannitol salt agar) were identified using Gram stain, catalase, and slide and tube coagulase tests. The isolates were confirmed as MRSA by disc diffusion (1 *μ*g oxacillin).

### 2.1. Drug Susceptibility Tests

The antimicrobial susceptibility of* S. aureus* isolates was assessed by disk-diffusion tests, according to the Clinical Laboratory Standards Institute guidelines [17, 18]. The following antimicrobial discs were used: oxacillin (1 *μ*g), ciprofloxacin (5 *μ*g), gentamicin (10 *μ*g), amikacin (30 *μ*g), linezolid (30 *μ*g), quinpristin-dalfopristin, cefoperazone (30 *μ*g), and vancomycin (30 *μ*g). All the discs were obtained from Hi-media (Hi-media, Mumbai, India).* S. aureus* ATCC 25923 was used as the internal control in each run of the test.

### 2.2. Data Collection

A standardized questionnaire was used to collect information on the risk factors for CA-MRSA colonization. The data collected were age, sex, educational degree, and marital status and whether the subject was living in a hostel or not, the number of household members, the presence of any household member who was a health care worker (HCW), the presence of any household member who was less than 7 years old, the presence of any household member who was bedridden, the presence of chronic diseases, smoking habits, hospitalizations within the previous year, a history of caring for inpatients within the past year, outpatient clinic visits within the past year, the use of antibiotics within the previous year, a history of skin and soft-tissue infection within the previous year, whether the subject takes a shower every day, and economic status.

### 2.3. Statistics

Statistical analysis of the risk factor association with SA and MRSA colonization was calculated using the Chi-square test (Epi info software).

## 3. Results

During the 2-month study period, there were 200 people enrolled.* Staphylococcus aureus* colonisation was found in 12% (SA = 24, *n* = 200) of participants. Methicillin resistant SA was identified in 5% (10/200). A total of 10 of these 24 people carried MRSA (41.66% of SA) and 14 had MSSA (58.33% of SA). The prevalence of SA and MRSA was analyzed against the surveyed factors (Table 1). One group demonstrated statistically significant correlation with SA: participants visiting OPD in the past year (*P* = 0.002). The prevalence of SA in healthcare workers was 13.33% (4/30). MRSA and MSSA were observed amongst 2 members each (MRSA 6.66%, MSSA 6.66%).

### 3.1. MRSA-Positive Individuals

Seven of the ten MRSA isolates were from females (Table 2). The mean age of those affected was 28.05 years. Six of the ten (6/10 = 60%) carriers reported previous contact with health-care facilities. Subjects who were MRSA-positive were compared with all other subjects and with subjects who were colonized with methicillin sensitive* S*.* aureus* with respect to previous hospital admission, recent antibiotic use, any contact with a health facility, and all other variables. No significant difference was found between MRSA-positive subjects and others using the chi-square test and Fisher's exact method.

**Figure 1:**
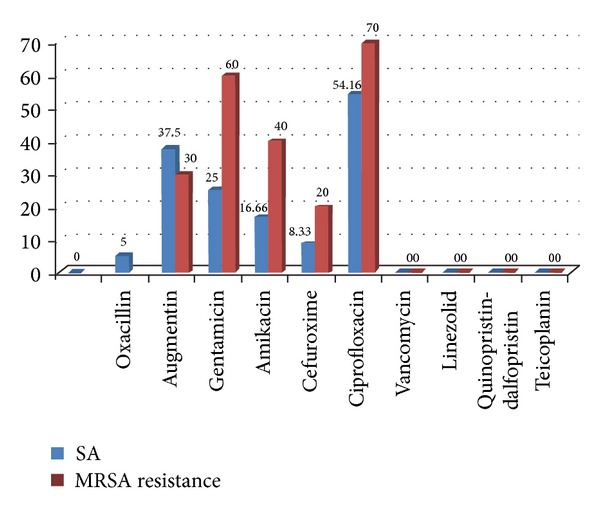
Comparative resistance of SA and MRSA.

Antibiotic susceptibility: Ciprofloxacin and Gentamicin showed 70% and 60% resistance towards MRSA, respectively. Vancomycin, Linezolid, and Quinupristin-dalfopristin were found to be 100% sensitive in the present study. Only one MRSA strain was found to be fully sensitive to all the drugs. No strain was found to be absolutely resistant to all drugs. (Table 2, Figure 1).

## 4. Discussion

Community-associated methicillin-resistant* Staphylococcus aureus* (MRSA) is emerging as a leading cause of skin and soft-tissue infections in many parts of the world [1]. Recently, many cases of MRSA infection have been reported in healthy community individuals with no traditional risk factors for MRSA infection [2].

*S. aureus* nasal carriage rate in the present study was found to be 12% (24/200). Usually quoted figures for* S. aureus* nasal carriage is 20–40% [19]. A study on healthy contacts of outpatient paediatric cases from an urban community at Delhi showed an alarming colonization rate of 5.3 percent [20]. A study from Japan reported 36% of* S. aureus* in nares of Japanese adults and 32.4% in nasal cavity of adults in USA, which is slightly higher in comparison to results of this study [21]. None of the carriers had any underlying conditions like diabetes or hypertension.

MRSA nasal carriage prevalence was found to be 5% (10/200) in the present study. Other community-based studies have also found low MRSA nasal carriage prevalence. The overall MRSA carriage rate of 8.5% was observed from South India [22]. Munckhof et al. found a prevalence of only 0.7% among 699 patients in Queensland, Australia [23]. A higher propensity was observed amongst MRSA strains reported in USA (24.15%) [24]. Taiwan and Zaria, Nigeria, reported a prevalence of 13.6% and 14.85%, respectively, from anterior nares of healthy population, adults and school pupils [25].

Seven of the ten MRSA isolates were from females, although it was not statistically significant (Table 2). A study from South India reported males to be the major carriers (15/118, 12.4%) [22]. The mean age of those affected was 28.05 years. Six of the ten (6/10 = 60%) carriers reported previous contact with health-care facilities.

Statistical analysis of the risk factor association with SA and MRSA colonization was calculated using the Chi-square test (Epi info software). Risk factors for MRSA colonization were identified by comparing people with MRSA colonization to those with MSSA colonization and people with MRSA colonization to those without carriage of* S. aureus* at the same time. This allowed us to avoid problems associated with multiple intergroup comparisons. The analysis indicated that visiting health-care facility in the past year was an independent risk factor for SA colonization compared to those without* S. aureus* colonization. Studies in the developed world support these findings and suggest that factors associated with CA-MRSA carriage include prior antibiotic usage, contact with health care facility, poor socioeconomic conditions, and overcrowding [22, 23].

During the study we also found that the prevalence of SA in healthcare workers was 13.33% (4/30) which is less as compared to that reported by another study from Delhi (37.3%) [26]. Studies elsewhere have reported 6–50% as carrier rate amongst health care workers, particularly those posted in the burns and intensive care units [22]. In the present study MRSA and MSSA were observed amongst 2 members each (MRSA 6.66%, MSSA 6.66%). These results are in consensus with findings by Goyal et al. (MRSA 6.6% among HCW) [26]. Prevention of MRSA infections merits discussion as once introduced in a hospital, MRSA is very difficult to eradicate [22]. Within hospital, MRSA spreads rapidly by hands of medical personnel. Colonised employees of hospital such as asymptomatic nasal and hand carriers acting as reservoirs are important sources for spreading this organism. Multiple, prolonged use of antibiotics and prolonged hospitalization are other important factors which make hospital an ideal place of transmission and perpetuation of MRSA.

The susceptibility test results showed ciprofloxacin to be the least effective agent with sensitivity being 54.16%. The present study shows ciprofloxacin resistance towards SA to be 60% which is in sharp contrast to the results observed by Chatterjee et al. with 2.4% [6].

Antibiotic sensitivity pattern of the MRSA isolates (Table 2) shows 70% resistance to ciprofloxacin (5 *μ*g) and 100% sensitivity to Vancomycin (30 *μ*g). These findings are consistent with a study from Delhi [26] where 60% MRSA were resistant to ciprofloxacin along with 100% sensitivity towards Vancomycin, but in sharp contrast to the results by Chatterjee et al. [6] with resistance to ciprofloxacin being 12.5%. None of the isolates were multidrug resistant in the present study unlike Goyal et al. who reported 30% (3/10) resistance to all the antibiotics tested [26, 27]. It is alarming to note that 60% (6/10) isolates were resistant to ciprofloxacin since it had been proposed as an alternative for treatment of MRSA infection [28]. Increasing number of resistant strains may be the result of selection pressure owing to uncontrolled drug usage in the community.

The survey described here has several limitations. Firstly, active surveillance using only specimens from the anterior nares to screen for MRSA colonization will detect fewer than 75% of carriers [21]. Nasal cultures may underestimate the real prevalence of* S. aureus* colonization, since it can also be found in other parts of the body as well, such as axilla and pharynx [7]. Specimens other than nasal swabs have been omitted from screening strategies for cost savings, and current recommendations do not emphasize their importance [19, 20].

Secondly, the present study's estimation of MRSA nasal carriers is based on the resistance to cefoxitin disc (30 *μ*g) and not on the detection of* mecA* gene that codes for the production of the altered penicillin binding protein (PBP2a) responsible for classical methicillin resistance. A study from Zaria, Nigeria, reported absence of* mecA* gene in MRSA isolates [29]. MRSA without* mecA* gene are also being implicated in the cause of some severe infections in communities and hospitals [29]. This emphasizes further study on the potentials of these MRSA isolates.

Lastly, these cases may represent carriage of hospital acquired strains in the community rather than transmission within the community. Further investigation is required to confirm this. This issue must be actively addressed to tackle the public health concern of widespread antibiotic resistance. The prevalence of* S. aureus* nasal carriage varies according to the quality of sampling, culture techniques, and the population studied [1, 6, 18].

## 5. Conclusion

In conclusion, the present study showed that the rate of* S. aureus* nasal carriage was found to be 12% (24/200) and MRSA nasal carriage prevalence was found to be 5% (10/200).

This study also identifies that visiting health-care facility in the past year was an independent risk factor for SA colonization. These findings could be helpful in controlling the spread of MRSA in community settings. We believe that MRSA surveillance studies in the community setting should be encouraged to gain a better understanding of clinical and molecular epidemiology of the emerging CA-MRSA isolates.

## Figures and Tables

**Table 1:** Calculated correlation between SA nasal and MRSA nasal colonization and various other surveyed factors.

Parameter	MRSA	MSSA	*P* value (MRSA)	*P* value (SA)
Sex				
Male	3	7	0.442	0.9736
Female	7	7	0.4063	0.89
Education				
Elementary school	8	9	0.141	0.171
Junior high school	2	5	0.141	0.171
Senior high school				
Marital status				
Married		6	15	0.466
Unmarried		4	9	0.3911
Presence of members <7 years of age				
Yes *n* = 84	3	9	0.442	0.427
Presence of bedridden members				
Yes *n* = 16	1	3	0.815	0.416
Chronically ill				
Yes *n* = 33	3	2	0.25	0.567
Smoking habits				
Yes	2	5	0.694	0.142
Shower everyday				
Yes *n* = 160	8	12	1	0.6831
Members who are HCW				
Yes *n* = 30	2	2	0.657	0.819

Factors related to exposure in the previous year				
Hospitalization				
Yes *n* = 53	3	4	0.802	0.767
Cared for in-patients				
Yes *n* = 53	3	4	0.802	0.767
Used antibiotic				
Yes *n* = 104	5	8	0.899	0.831
Visited OPD				
Yes *n* = 53	4	9	0.333	0.0021
Skin/soft tissue injury				
Yes *n* = 54	4	5	0.354	0.246

**Table 2:** Antibiotic resistance towards SA and MRSA.

Antibiotics	SA resistance	MRSA resistance
Oxacillin	5	5
Augmentin	37.5	30
Gentamicin	25	60
Amikacin	16.66	40
Cefuroxime	8.33	20
Ciprofloxacin	54.16	70
Vancomycin	0	0
linezolid	0	0
Quinupristin-dalfopristin	0	0
Teicoplanin	0	0
